# Lactobacillus rhamnosus Infective Endocarditis in an Elderly Male

**DOI:** 10.7759/cureus.47481

**Published:** 2023-10-22

**Authors:** Yazeed G Sweedan, Sidra Kalsoom, Muhammad A Zaman, Chuong Le, Latashu Naidu

**Affiliations:** 1 Internal Medicine, Conemaugh Memorial Medical Center, Johnstown, USA; 2 Infectious Disease, Conemaugh Memorial Medical Center, Johnstown, USA

**Keywords:** transesophageal echocardiogram, transthoracic echocardiogram, daptomycin, infective endocarditis, lactobacillus rhamnosus

## Abstract

Lactobacilli are facultative anaerobic, gram-positive, rod-shaped bacteria found in the normal flora of the oral cavity and the gastrointestinal and genitourinary tracts. This report presents a case of *Lactobacillus rhamnosus* infective endocarditis and provides echocardiographic evidence of its pathogenic potential. Furthermore, we provide an account of the first successful treatment with daptomycin to our knowledge. Additionally, we examine the limited literature available on this microbiological entity and attempt to relate this data to our case.

## Introduction

Lactobacilli are facultative anaerobic, gram-positive, rod-shaped bacteria found in the normal flora of the oral cavity and the gastrointestinal and genitourinary tracts [1]. The previously perceived lack of pathogenicity of some *Lactobacillus* spp. strains, along with their beneficial effect on the gut microflora and subsequent prevention of diarrhea, have led to their frequent usage as probiotic products [2]. However, with numerous cases of *Lactobacillus* infections recently documented in the literature, their pathogenicity and ability to induce severe infections in humans have been recognized [1]. More than 100 *Lactobacillus* endocarditis cases have been described in the literature [1]. Along with infective endocarditis, lactobacilli have been reported to cause dental caries, meningitis, splenic abscesses, endometritis, and chorioamnionitis [1]. This report presents a case of *Lactobacillus rhamnosus* infective endocarditis with a 10 mm mitral valve vegetation detected on transesophageal echocardiogram, which was successfully treated with a six-week course of daptomycin.

## Case presentation

A 65-year-old male with multiple neurological disabilities stemming from a childhood anoxic brain injury was brought from his long-term care facility to the emergency department for increased work of breathing noticed by the nursing staff. The patient’s disabilities included intellectual disability, mutism, blindness, dysphagia with a percutaneous gastrostomy tube (PEG) tube dependence, paraparesis, severe contractures, and thoracolumbar scoliosis. The patient’s past medical history included benign prostatic hyperplasia and sigmoid volvulus leading to sigmoid colectomy with subsequent abdominal adhesions and recurrent episodes of small bowel obstruction. The emergency medical services reported pulse oximetry readings of 84% on room air at the care facility, which improved to 100% after initiating oxygen supplementation with a nasal cannula. In the emergency department, the patient was afebrile and normotensive, but tachycardic and tachypneic. His pulse oximetry reading remained at 100% on 3 L of oxygen supplementation. Physical examination was remarkable for the absence of teeth/dentures, reducible periumbilical hernia, and normal bowel sounds, but negative for signs of infective endocarditis, including Osler nodes, Janeway lesions, petechiae, conjunctival hemorrhages, or splinter hemorrhages.

Initial laboratory testing was remarkable for leukocytosis with neutrophilic predominance, normocytic anemia, elevated total protein, and hypoalbuminemia (Table 1). Serum inflammatory markers including erythrocyte sedimentation rate, C-reactive protein, and procalcitonin were elevated (Table 1). Coronavirus disease 2019 polymerase chain reaction and rapid influenza A/B antigen testing were both negative. Urinalysis was unremarkable. Two sets of blood cultures from two separate venipuncture sites were acquired as part of the sepsis workup.

**Table 1:** Serum laboratory test results during the hospital stay.

	Reference range	Emergency department	Day one	Day two	Day three	Day four	Day five	Day six	Day seven
White blood cell count	3.1–8.5 10^3^/µL	10.34	8.52	7.42	7.0	6.42	6.45	7.28	6.68
Neutrophils	38.0–70.0%	75.8	71.2	70.7	66.5	61.7	68	60	68
Lymphocytes	20.0–48.0%	13.2	14.4	15.1	18.6	23	18	21	19
Red blood cell count	4.5–6.3 10^6^/µL	3.34	3.04	2.92	3.0	3.06	3.15	3.18	3.34
Hemoglobin	14–18 g/dL	9.3	8.4	8.1	8.2	8.5	8.9	8.9	9.2
Hematocrit	40–54%	30	28	27	27	28	29	29	31
Mean corpuscular volume	82–101 fL	90	93	91	91	92	91	92	92
Mean corpuscular hemoglobin	27.0–34.0 pg	27	27	27	27	27.8	28	28	27.5
Red cell distribution width	11.5 - 14.5 %	14.8	14.8	14.8	14.8	14.6	14.5	14.6	14.8
Platelet count	140–440 10^3^/µL	267	245	242	243	263	247	265	260
Sodium	136–145 mmol/L	138	142	141	142	138	138	137	138
Potassium	3.5–5.1 mmol/L	4.8	4.3	4.1	4.3	4.3	4.4	4.5	4.5
Chloride	98–107 mmol/L	106	108	108	108	106	106	105	104
Bicarbonate	23–31 mEq/L	27	28	27	27	26	27	26	27
Calcium	8.5–10.3 mg/dL	8.6	8.6	8.5	8.6	8.8	8.8	9.1	9.1
Magnesium	1.6–2.6 mg/dL	1.6	1.7	1.5	1.7	1.5	1.6	1.6	1.7
Phosphorus	2.3–4.7 mg/dL	3.8	-	3.3	3.5	3.3	3.4	4.1	3.7
Blood urea nitrogen	8–26 mg/dL	24	23	22	22	18	17	17	18
Creatinine	0.55–1.30 mg/dL	1.0	1.0	1.0	1.0	0.9	0.9	0.9	1.0
Estimate glomerular filtration rate	>60 mL/minute	84	84	84	84	95	95	95	84
Total bilirubin	0.3–1.2 mg/dL	0.2							
Aspartate aminotransferase	5-34 U/L	14							
Alanine aminotransferase	≤55 U/L	16							
Alkaline phosphatase	56–119 U/L	65							
Total protein	g/dL	9.4							
Albumin	g/dL	2.7							
Erythrocyte sedimentation rate	0–20 mm/hour	>140							
C-reactive protein	0–0.8 mg/dL	3.0							
Procalcitonin	0–0.05 ng/mL	0.14							
Rheumatoid factor	-	Negative							
High-sensitivity troponin I	0–53 ng/L	18.06							
B-type natriuretic peptide	0–100 pg/mL	63							

An electrocardiogram demonstrated sinus tachycardia, while high-sensitivity troponin remained within normal limits. Computed tomography angiography of the chest showed left lower lobe ground-glass opacities, a left lung base calcified granuloma, right upper lobe emphysematous bulla, and a 2 mm lung nodule in the right lung base. Contrast-enhanced computed tomography (CT) of the abdomen and pelvis demonstrated multiple hypodense lesions in the liver (largest diameter was 1 cm), bilateral non-obstructing renal calculi, bilateral renal cysts (largest diameter was 2.6 cm), supraumbilical hernia containing a segment of the transverse colon, and a mildly enlarged prostate with calcifications. The patient was continued on 3 L oxygen supplementation by a nasal cannula for acute hypoxic respiratory failure. Sepsis management with intravenous (IV) fluid restriction and broad-spectrum empiric antibiotics (vancomycin and ceftriaxone) was initiated. Finally, the patient was admitted to the medical floor for further evaluation and management.

During the first three days of hospital admission, the patient remained afebrile and his white blood cell count normalized. The two sets of blood cultures grew Gram-positive rods in all four bottles (two aerobic and two anaerobic bottles) after 72 hours, which were later identified as *L. rhamnosus*. Infectious disease was brought on board and proceeded to switch the patient’s empiric antibiotic coverage to a targeted antibiotic regimen with daptomycin at 6 mg/kg/day.

**Figure 1:**
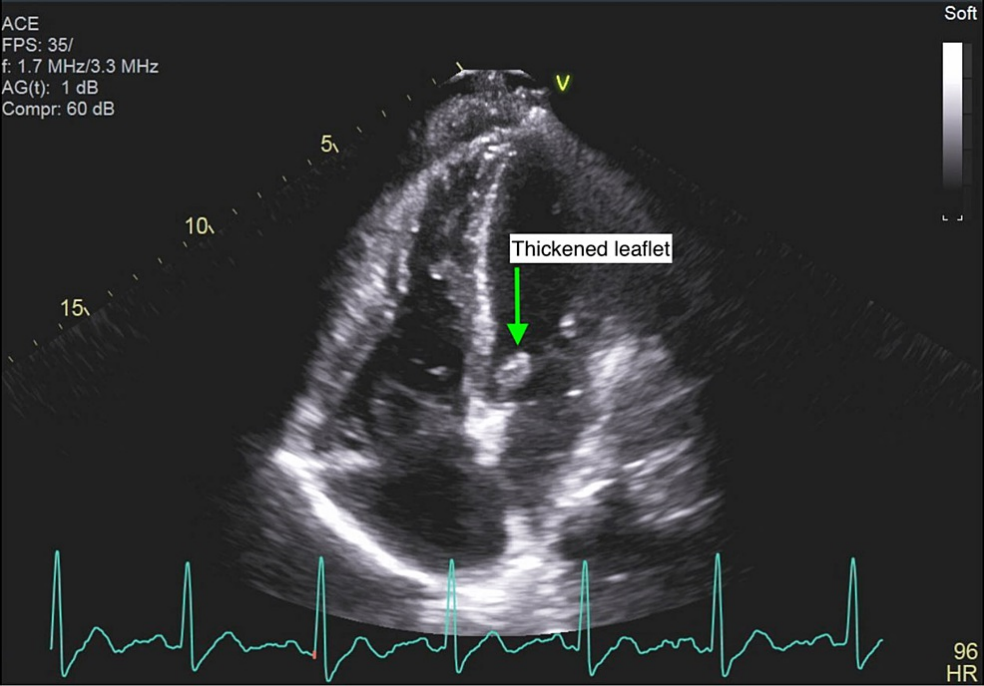
Transthoracic echocardiogram showing thickening of the posterior leaflet subvalvular apparatus of the mitral valve.

**Figure 2:**
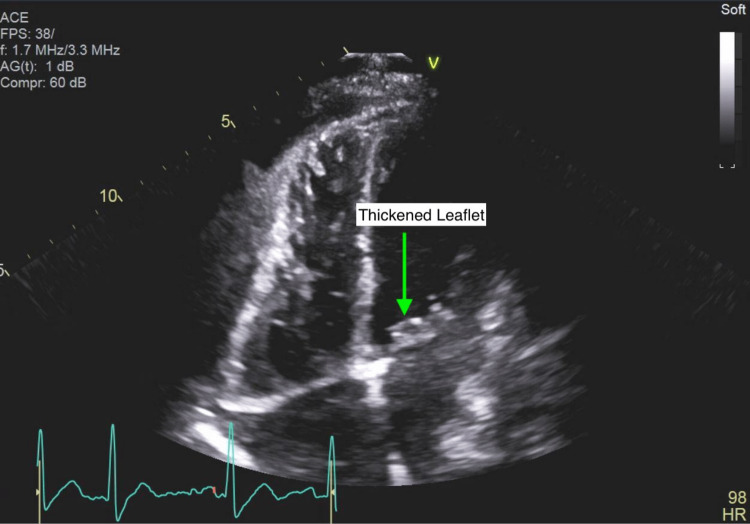
Transthoracic echocardiogram showing thickening of the posterior leaflet subvalvular apparatus of the mitral valve.

**Figure 3:**
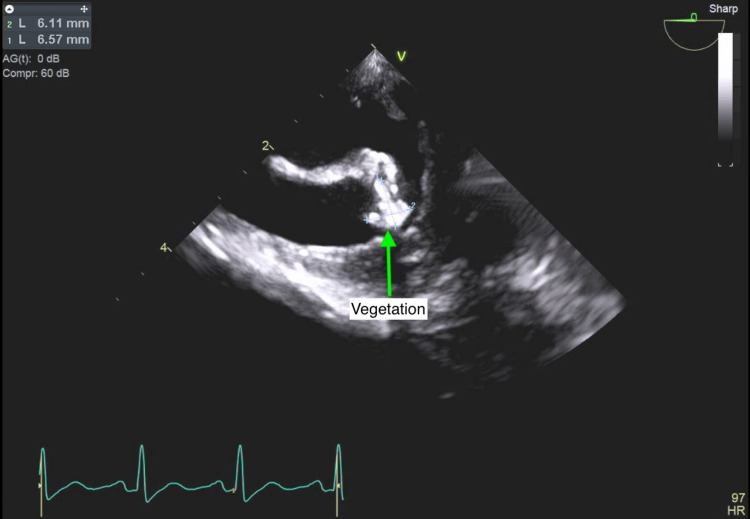
Transesophageal echocardiogram showing a 10 mm echodensity attached to the anterior mitral valve leaflet.

**Figure 4:**
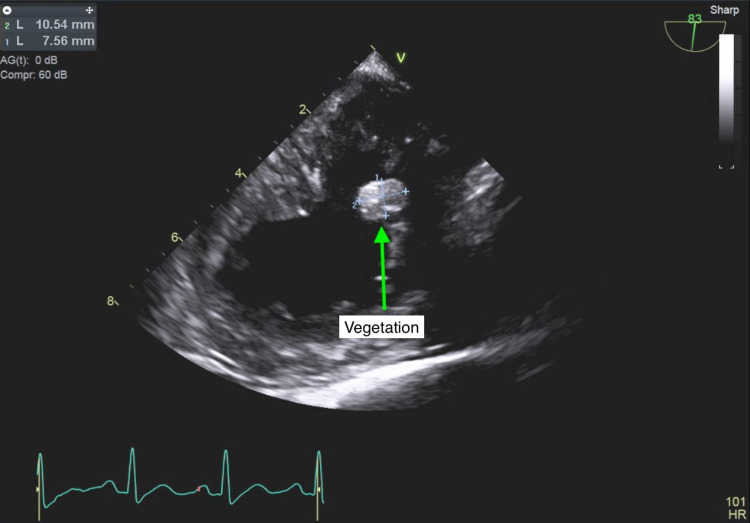
Transesophageal echocardiogram showing a 10 mm echodensity attached to the anterior mitral valve leaflet.

**Figure 5:**
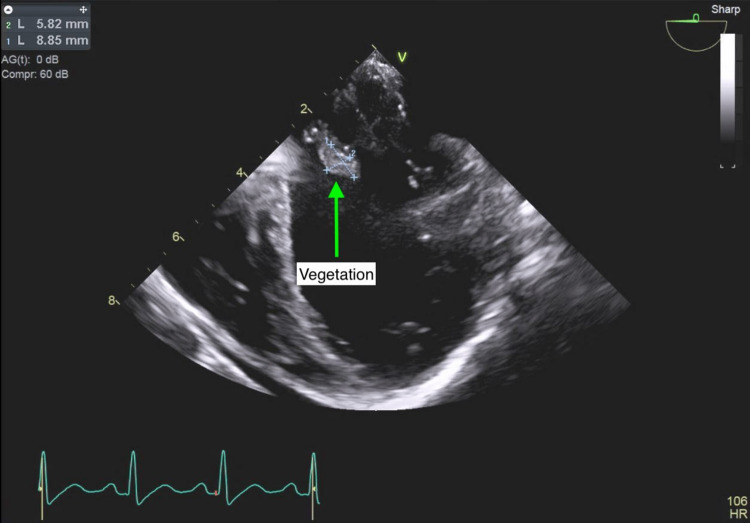
Transesophageal echocardiogram showing a 10 mm echodensity attached to the anterior mitral valve leaflet.

Cardiology was brought on board for echocardiographic assessment of possible infective endocarditis. A transthoracic echocardiogram was performed on the fourth day of hospitalization but was technically difficult to interpret due to rib shadowing. The limited data that were obtained included a hyperdynamic left ventricle with an ejection fraction of 70%, a small circumferential pericardial effusion, and thickening of the posterior leaflet of the mitral valve (Figures 1, 2). However, proper assessment of the heart valves was not possible. A transesophageal echocardiogram (TEE) was performed a couple of days later but was technically difficult due to contractures in the neck with severe rotation of the esophagus and heart. However, a 10 mm echodensity attached to the anterior mitral valve leaflet was detected, which exhibited independent motion (Figures 3-5). There also appeared to be mitral regurgitation of moderate severity. A cardiac CT with functional assessment was performed the next day, which demonstrated an abnormally thickened anterior mitral valve leaflet.

Cardiothoracic surgery was consulted for evaluation of possible surgical intervention of the sub-centimeter mitral valve vegetation. In light of the patient’s complex medical comorbidities along with the size of the mitral vegetation and absence of evidence of embolism, surgical excision was deemed to provide very little advantage and would most likely require valve replacement. As such, they recommended aggressive medical management followed by a repeat echocardiogram in approximately one month for the re-evaluation of possible surgical intervention. As such, interventional radiology placed a 4-French, single lumen, 35 cm long Navilyst Power PICC via the basilic vein with its tip at the cavo-atrial junction. The patient was discharged on IV daptomycin at 8 mg/kg/day (540 mg IV Q 24 hours) for six weeks from the first negative blood culture. Repeat TEE was scheduled in one month to assess response to antibiotics.

## Discussion

A literature review published in 2020 examined all reported cases of Lactobacillus endocarditis since 1992 (50 cases) [3]. The review reported the mean age of patients to be 52.4 years and found the predominant gender to be male at 68% [3]. Valvopathy was reported in 64% of patients and appeared to be the most common underlying condition [3]. A history of dental procedures was found in 34% of cases, while 22% of cases had a history of prior invasive procedures [3]. Overall, 22% of patients reported using probiotics, and 16% suffered from immunosuppression [3]. Similar to infective endocarditis as a whole, the most common symptom was found to be fever at 72%, while a new murmur was found to be the most common physical examination finding at 30% [3]. Both the aortic and mitral valves were affected in the same proportion, while only two cases involved the tricuspid valve [3]. Furthermore, 14% of endocarditis occurred on a prosthetic valve [3]. *L. Rhamnosus* was the most commonly isolated species (24%) followed by *L. acidophilus* (18%), *L. paracasei* (12%), and *L. casei* (12%) [3]. According to the antibiotic susceptibility data that was available in 26 studies, lactobacilli was found to be highly sensitive to penicillin G (86%), amoxicillin (86%), ampicillin (83%), clindamycin (100%), rifampin (90%), and gentamicin (79%) [3]. However, susceptibility to vancomycin and cefotaxime was low at 21% and 43% of cases, respectively [3]. The mean and median length of the administered antibiotic courses were 44.7 days and 42 days, respectively [3]. Surgery was required in half of the cases (54%) [3]. The mortality rate was found to be as high as 10% [3]. The study concluded that high-dose penicillin, combined with an aminoglycoside, should be recommended as first-line, while clindamycin should be preferred over vancomycin in penicillin-allergic patients [3].

In 2022, a survey of all the case reports of lactobacilli infections between 2019 and 2021 was published, which found endocarditis to be the predominant manifestation in 23 of 48 cases [4]. A recent increase in incidence was detected and assumed to be mostly related to the increased consumption of probiotics and a higher percentage of predisposing conditions within the population [4]. Most of these endocarditis cases had a slow onset, with or without fever [4]. Valvular vegetations became detectable after many days or even months from the onset of symptoms [4]. Even though antibiotic treatment was successful in all cases, many still required valvular replacement [4]. Susceptibility to β-lactams, including amoxicillin, amoxicillin/clavulanic acid, ampicillin, ampicillin-sulbactam, benzylpenicillin, penicillin G, piperacillin/tazobactam, and meropenem, was reported in most cases, which fell in line with what was reported previously for lactobacilli (intrinsic high level of resistance to vancomycin and susceptibility to β-lactams) [4]. Resistance of lactobacilli to cephalosporins appeared to be a common trait [4]. Finally, the survey concluded that the ability of lactobacilli to behave as a pathogen is inherent to the strain or clone, with the most relevant virulence factor being the capacity to form a biofilm [4]. Strikingly, this trait varied between clones of the same probiotic *L. rhamnosus* GG [4].

Our patient was of the gender reported to predominate in lactobacilli endocarditis cases but was older than the reported mean age [3]. Of his expansive comorbid conditions, dysphagia with PEG dependence may have been the most important predisposing factor. However, in light of metagenomic studies and the concept of the super-organism, where functioning is based on cooperation between microbiota and the human body, explaining the translocation process might be much more difficult than expected [2]. Detailed investigation of possible probiotic usage in the nursing home was negative, making it an unlikely predisposing factor [3]. The patient also lacked teeth/dentures or any history of recent dental procedures making an oral source unlikely [3]. Unfortunately, antibiotic sensitivity was not performed due to a laboratory error. However, treatment with a six-week course of daptomycin proved to be successful in alleviating the fever, normalizing the inflammatory markers, preventing further blood culture growth, and returning the patient back to his baseline. The repeat TEE was not performed due to the patient’s hemoglobin gradually dropping to 5.3 g/dL without any signs of bleeding. A blood transfusion was not performed based on the patient’s religious beliefs. Fortunately, the patient’s hemoglobin did improve with the administration of epoetin alfa and iron.

## Conclusions

We present this case to raise awareness of the pathogenic potential of *L. rhamnosus* and the importance of further evaluation for possible infective endocarditis after its detection in blood cultures. To our knowledge, we also report the first case of successful treatment of *L. rhamnosus* infective endocarditis with daptomycin to further expand the list of effective antibiotics for this specific bacteria. Finally, we agree with previous articles that have advocated for the need for further research on this microbiological entity, especially when it is being utilized in mass-produced, over-the-counter consumer products such as probiotics.
